# The level and distribution of the GABA_B_R1 and GABA_B_R2 receptor subunits in the rat's inferior colliculus

**DOI:** 10.3389/fncir.2012.00092

**Published:** 2012-11-26

**Authors:** Lena Jamal, Aziz N. Khan, Sehrish Butt, Chirag R. Patel, Huiming Zhang

**Affiliations:** Department of Biological Sciences, University of WindsorWindsor, ON, Canada

**Keywords:** hearing, auditory system, auditory midbrain, GABA, GABA_B_ receptor, GABA_B_R1 subunit, GABA_B_R2 subunit, inhibition

## Abstract

The type B γ-aminobutyric acid receptor (GABA_B_ receptor) is an important neurotransmitter receptor in the midbrain auditory structure, the inferior colliculus (IC). A functional GABA_B_ receptor is a heterodimer consisting of two subunits, GABA_B_R1 and GABA_B_R2. Western blotting and immunohistochemical experiments were conducted to examine the expression of the two subunits over the IC including its central nucleus, dorsal cortex, and external cortex (ICc, ICd, and ICx). Results revealed that the two subunits existed in both cell bodies and the neuropil throughout the IC. The two subunits had similar regional distributions over the IC. The combined level of cell body and neuropil labeling was higher in the ICd than the other two subdivisions. Labeling in the ICc and ICx was stronger in the dorsal than the ventral regions. In spite of regional differences, no defined boundaries were formed between different areas. For both subunits, the regional distribution of immunoreactivity in the neuropil was parallel to that of combined immunoreactivity in the neuropil and cell bodies. The density of labeled cell bodies tended to be higher but sizes of cell bodies tended to be smaller in the ICd than in the other subdivisions. No systematic regional changes were found in the level of cell body immunoreactivity, except that GABA_B_R2-immunoreactive cell bodies in the ICd had slightly higher optic density (OD) than in other regions. Elongated cell bodies existed throughout the IC. Many labeled cell bodies along the outline of the IC were oriented in parallel to the outline. No strong tendency of orientation was found in labeled cell bodies in ICc. Regional distributions of the subunits in ICc correlated well with inputs to this subdivision. Our finding regarding the contrast in the level of neuropil immunoreactivity among different subdivisions is consistent with the fact that the GABA_B_ receptor has different pre- and postsynaptic functions in different IC regions.

## Introduction

γ-aminobutyric acid (GABA) is an important inhibitory neurotransmitter in the central nervous system (Enna and Möhler, 2007). This neurotransmitter exists at a high level in the midbrain auditory structure, the inferior colliculus (IC) (Roberts and Ribak, 1987; Merchán et al., 2005). Neurons in the IC receive GABAergic projections from extrinsic sources as well as local inhibitory interneurons (Adams and Mugnaini, 1984; Helfert et al., 1989; Li and Kelly, 1992; Vater et al., 1992; Shneiderman et al., 1993; Merchán et al., 1994; González-Hernández et al., 1996; Zhang et al., 1998; Kulesza and Berrebi, 2000; Riquelme et al., 2001; Saldaña et al., 2009). GABAergic receptors in the IC include the metabotropic GABA_B_ receptor as well as the ionotropic GABA_A_ receptor (Glendenning and Baker, 1988; Marianowski et al., 2000; LeBeau et al., 2001; Shiraishi et al., 2001; Zhang and Kelly, 2003; Malmierca and Merchán, 2004; Kelly and Caspary, 2005; Hilbig et al., 2007; Caspary et al., 2008; Jamal et al., 2011).

The GABA_B_ receptor contributes to sound-driven responses in the IC (Faingold et al., 1989; Szczepaniak and Møller, 1995, 1996; Vaughn et al., 1996; Burger and Pollak, 1998). These contributions are dependent on the pre- and/or postsynaptic functions of the receptor (Zhang and Wu, 2000; Ma et al., 2002; Sun et al., 2006; Sun and Wu, 2009). Activation of presynaptic GABA_B_ receptors reduces the release of neurotransmitters including glutamate and GABA (Ma et al., 2002; Sun et al., 2006). This reduction is a result of decreased calcium influx (Mintz and Bean, 1993; Filippov et al., 2000; Kornau, 2006; Ulrich and Bettler, 2007). Activation of postsynaptic GABA_B_ receptors leads to prolonged membrane hyperpolarization (Sun and Wu, 2009). This membrane-voltage change is due to an increase in the opening probability of potassium channels (Luscher et al., 1997; Ulrich and Bettler, 2007). The receptor also contributes to long-term enhancement of excitatory neural responses in the IC (Zhang and Wu, 2000).

A functional GABA_B_ receptor is a heterodimer consisting of two subunits, GABA_B_R1 and GABA_B_R2 (Huang, 2006). Both of these subunits are made in the endoplasmic reticulum. Due to a retention signal, the GABA_B_R1 subunit remains within the endoplasmic reticulum after it is made. Binding by the GABA_B_R2 subunit masks the retention signal, allowing the two subunits to form a heterodimer and to traffic toward the plasma membrane (Pin et al., 2004; Pooler and McIlhinney, 2007).

The GABA_B_ receptor is not homogeneously expressed in the IC. Receptor autoradiographic studies have revealed that functional GABA_B_ receptors are more abundant in the dorsomedial than the ventral region of the structure (Milbrandt et al., 1994; Fubara et al., 1996; Hilbig et al., 2007). Our recent immunohistochemical study on the GABA_B_R2 subunit revealed a similar distribution in the rat's IC (Jamal et al., 2011).

It has yet to be determined whether the GABA_B_R1 subunit has a similar distribution in the IC. Also, it is important to find how the level of the GABA_B_ receptor in the IC is dependent on the density of cell bodies expressing the receptor and the abundance of the receptor in the neuropil. Furthermore, it is important to examine the morphological features of cells expressing the receptor. Addressing these questions can provide an insight into the role of the receptor in auditory processing. Therefore, we conducted Western blotting and immunohistochemical experiments to examine the expression of the GABA_B_R1 and GABA_B_R2 subunits in the IC.

## Experimental procedures

### Animal preparation

Experiments were conducted using 11 male adult Wistar albino rats (*Rattus norvegicus*). These rats had a body weight of 250–400 g and were obtained from Charles River Canada Inc., St. Constant, Quebec. The animals were housed in the University of Windsor animal care facility for at least a week before experiments were conducted. The noise level in the animal facility was 55–60 dB SPL. All experimental procedures were approved by the University of Windsor Animal Care Committee and were in accordance with the guidelines of the Canadian Council on Animal Care.

### Western blotting

For each experiment, an animal was euthanized by an overdose of sodium pentobarbital (120 mg/kg, i.p.). The brain was extracted and sliced in the coronal plane into 240 μm thick sections using a VT1000S vibratome (Leica Microsystems, Heidelberg, Germany). Tissues of the central nucleus, the dorsal cortex, and the external cortex of the IC (ICc, ICd, and ICx) were collected from the resulting brain slices using a scalpel blade and an SZX7 stereoscope (Olympus, Tokyo, Japan). The IC was subdivided based on a standard rat brain atlas (Paxinos and Watson, 2007) and current anatomical results on this structure (Malmierca et al., 1993, 1995, 2011; Oliver, 2005; Loftus et al., 2008). The lateral and the rostral cortices of the IC as suggested by recent publications (Loftus et al., 2008; Malmierca et al., 2011) were combined into an external cortex in the present study.

For Western blotting analysis, a sample of a subdivision of the IC was formed by combining tissue from all the different slices with the subdivision. The entire cerebellum and a part of the liver were also collected and used as controls. Thus, a set of five samples was formed for Western blotting analyses for each independent case (i.e., each individual animal). During slicing and tissue collection, the brain was submerged in artificial cerebrospinal fluid containing (in mM): 126 NaCl, 3 KCl, 1.4 KH_2_PO_4_, 26 NaHCO_3_, 4 glucose, 1.3 MgSO_4_, and 1.4 CaCl_2_.

Tissue in each sample was homogenized manually in homogenization buffer (0.32 M sucrose in 5 mM Tris, pH 7.4) containing protease inhibitors (3 μM aprotinin, 10 μM phenylmethyl sulfonyl fluoride, 1 μM leupeptin, and 3 μM pepstatin). Lysate was cleared at 3400 g for 20 min at 4°C. The protein concentration of the supernatant was measured using a Bradford assay (Sigma-Aldrich, Oakville, ON) and quantified using a Biomate5 spectrophotometer (Thermo Scientific, Surrey, United Kingdom).

Thirty micrograms of protein from each sample were added to 4X sample buffer and subjected to electrophoresis on a 10% sodium dodecyl sulphate-polyacrylamide gel (SDS-PAGE) for 2 h at 125 V. Proteins were transferred from the gel to a polyvinylidene fluoride (PVDF)-Plus 0.45 μm membrane (Osmonics Inc., Minnetonka, MN) for 2 h at 30 V. The membrane was blocked at room temperature for 1 h in Tris-Buffered Saline Tween (TBST, 50 mM Tris/HCl, 153 mM NaCl, 0.05% Tween-20, pH 7.6) containing 1% skim milk. The membrane was then incubated in a primary antibody (see section “Antibodies and Control Experiments”) overnight at 4°C. Following three TBST washes (10 min each), the membrane was incubated in a secondary antibody (see section “Antibodies and Control Experiments”) for 1 h at room temperature. Following another three TBST washes (10 min each), the membrane was developed with an ECL kit (Pierce, Rockford, IL). Images were acquired using an HD2 gel imaging system and AlphaEase digital analysis software (Alpha Innotech, San Leandro, CA).

### Immunohistochemistry

A rat was euthanized by an overdose of sodium pentobarbital (120 mg/kg, i.p.) and transcardially perfused with Tyrode's solution followed by 4% paraformaldehyde in 0.1 M PB. The brain was extracted and cryoprotected in a sucrose gradient (10, 20, and 30% in 0.1 M PB) at 4°C. The brain was then sectioned into 30 μm slices in the coronal plane using a CM1050 S cryostat (Leica Microsystems, Heidelberg, Germany) and thaw-mounted onto SuperFrost Plus glass slides (Fisher Scientific, Pittsburg, PA). Every fourth section over the entire rostrocaudal extent of the IC was collected to form a set of tissue samples. Out of the four sets of samples, one or two were used for the present study (see section “Results”). The other sets were used for purposes not related to this study. For an immunoreaction, each step was conducted with all the sections in a set placed in a single container (keeper), so that the same experimental conditions were applied to the entire set of sections.

Prior to an immunoreaction, sections were warmed to room temperature. They were then incubated overnight at room temperature in a primary antibody (see section “Antibodies and Control Experiments”) in 0.1 M PBS with 0.05% Triton X-100 and 5% normal donkey serum (Jackson ImmunoResearch Laboratories, 017-000-121). Following three thorough washes with 0.1 M PBS (10 min each), the sections were incubated in a secondary antibody (see section “Antibodies and Control Experiments”) in 0.1 M PBS containing 2% normal donkey serum at room temperature for 2 h. After three additional washes (10 min each) in 0.1 M PBS, sections were incubated in ExtrAvidin®-peroxidase (Sigma E2886, 1:400) in 0.1 M PBS for 1.5 h at room temperature. The sections were then rinsed three times (10 min each) and incubated in 0.05% 3, 3-Diaminobenzidine tetrahydrochloride (DAB) in 0.1 M PB with 0.04% NiSO_4_ and 0.1% glucose oxidase at room temperature for 15–30 min. The DAB reaction was terminated by a wash with 0.1 M PBS. The tissues were then dehydrated with an ethanol gradient (60, 70, 95, 100, and 100%) and cleared twice with Histosol (10 min each). The slides were mounted with Permount (Fisher Scientific, SP-500) and coverslipped. Sections were examined using a *CTR 6500* microscope (Leica Microsystems, Heidelberg, Germany) and photomicrographic images were taken using a DFC 380 FX digital camera (Leica Microsystems, Heidelberg, Germany).

### Antibodies and control experiments

The primary antibody for probing the GABA_B_R1 subunit in both Western blotting and immunohistochemical experiments was rabbit polyclonal GABA_B_R1 antiserum (Santa Cruz Biotechnology R-300, 1:3000 for Western blotting and 1:1000 for immunohistochemistry). The primary antibody for probing the GABA_B_R2 subunit in Western blotting and immunohistochemical experiments was guinea-pig polyclonal GABA_B_R2 antiserum (Chemicon AB5394, 1:3000 for Western blotting and 1:1000 for immunohistochemistry). Primary antibodies for probing Actin and α-Tubulin in Western blotting experiments were mouse monoclonal anti-Actin antiserum (Chemicon MAB1501, 1:1000) and mouse monoclonal anti-α-Tubulin antiserum (Chemicon 05-829, 1:1000), respectively.

Secondary antibodies used in Western blotting experiments were horseradish peroxidase (HRP)-conjugated Goat anti-rabbit IgG (Santa Cruz Biotechnology SC-2004, 1:6000), HRP-conjugated goat anti-guinea pig IgG (Chemicon AQ108, 1:6000), and HRP-conjugated goat anti-mouse IgG (Chemicon 12-349, 1:10000). Secondary antibodies used in immunohistochemistry experiments were biotinylated donkey anti-rabbit IgG (Jackson ImmunoResearch Laboratories 711-005-152, 1:400) and biotinylated donkey anti-guinea pig IgG (Jackson ImmunoResearch Laboratories 706-065-148, 1:400).

**Figure 1:**
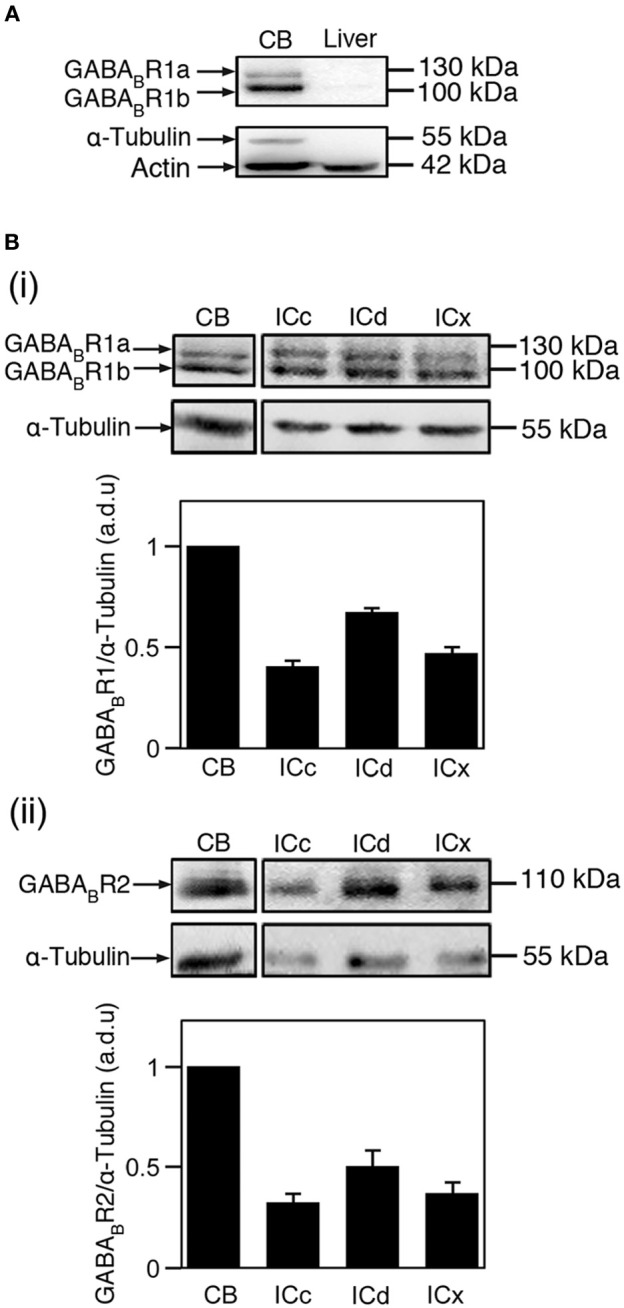
**Immunoreactivity to antibodies against the GABA_B_R1 and GABA_B_R2 subunits as revealed by Western blots**. **(A)** Western blots obtained by using the antibody against the GABA_B_R1 subunit and tissues from the cerebellum and the liver (top panel). Actin was used as a general loading control (lower band of the lower panel) and α-Tubulin was used as a brain tissue-specific loading control (upper band of the lower panel). Two bands with molecular weights of 100 and 130 kDa are revealed in the blot for cerebellar tissue. **(B)** Western blots showing GABA_B_R1 **(i)** and GABA_B_R2 **(ii)** immunoreactivities in the cerebellum, ICc, ICd, and ICx. In **(i)** and **(ii)**, blots reflecting α-Tubulin immunoreactivity are shown below the blots reflecting the GABA_B_R1 and GABA_B_R2 immunoreactivities. The ratio between the OD of a GABA_B_R1 or GABA_B_R2 band and the OD of an α-Tubulin band was obtained for each of the four neural structures in each animal. The ratio from the cerebellum was used for normalization. Group results based on ratios from four animals are shown in bar charts in **B(i)** and **B(ii)**. Error bars indicate standard errors.

**Figure 2:**
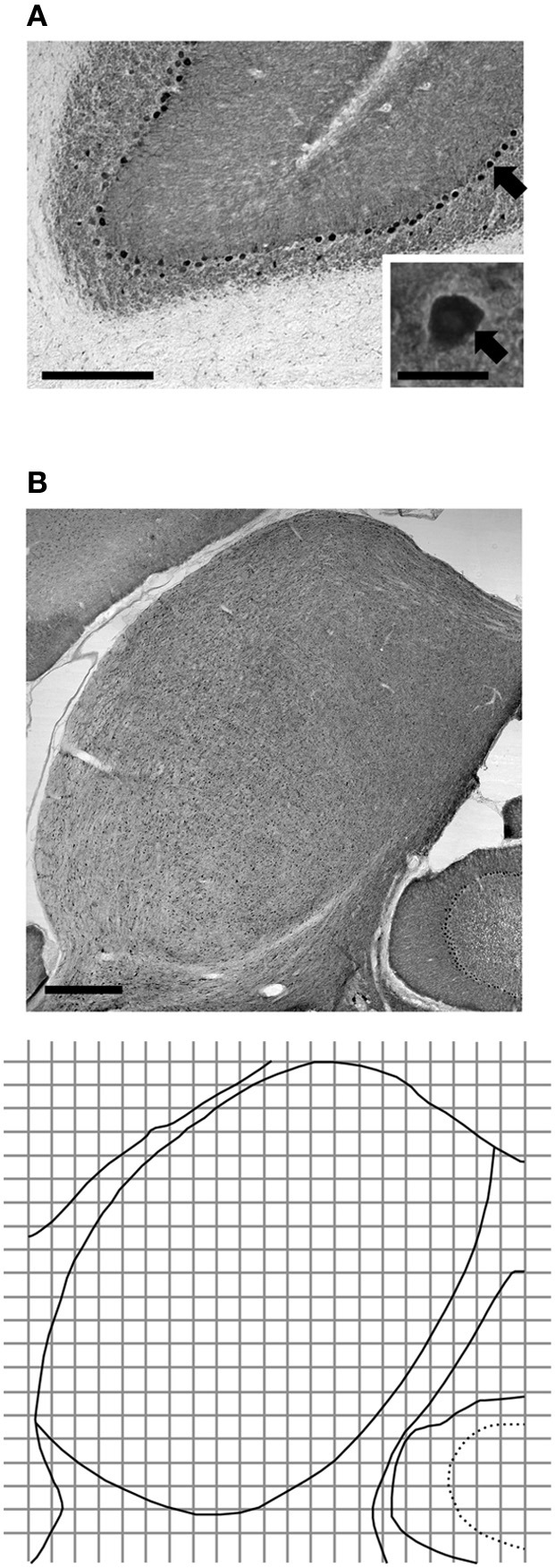
**Immunoreactivity to the antibody against the GABA_B_R1 subunit in the cerebellum (A) and the IC (B) as revealed by immunohistochemistry**. The cerebellar section shows molecular, Purkinje cell, and granule cell layers, and white matter. Inset in **(A)** shows a labeled somata of a Purkinje cell, as well as adjacent areas in the molecular and granule layers. Arrow points toward the labeled Purkinje cell. The diagram in the bottom panel of **(B)** is the outline of the section shown in the top panel of **(B)** along with a grid used in the measurement of cell body and neuropil immunoreactivity. The grid was also used in the measurement of morphological features of immunoreactive cells. Each grid box has 150 × 150 μm dimensions. Scale bars in **(A)** and **(B)**: 500 μm in low magnification image; 25 μm in the inset.

The effectiveness and specificity of the antibody against the GABA_B_R2 subunit had been verified by our previous Western blotting and immunohistochemical experiments (Jamal et al., 2011) and were confirmed by control experiments in the present study. In agreement with previous findings (Charles et al., 2001; Benke et al., 2002; Panzanelli et al., 2004), our Western blotting experiments using the antibody against the GABA_B_R1 subunit and cerebellar tissue revealed two bands at 100 and 130 kDa, respectively, (Figure 1A). These bands were absent in the lane for liver tissue. Further experiments using antibodies against Actin and α-Tubulin revealed that loading was even, and that α-Tubulin can serve as a selective loading control for neural tissue. Immunohistochemical experiments using cerebellar tissue revealed labeling by the antibody against the GABA_B_R1 subunit in the molecular layer, Purkinje cell layer, and granule cell layer (Figure 2A). Immunoreactivity was absent in white matter. No labeling was found in the cerebellum and the IC when the primary antibody was replaced by 0.1 M PBS (data not shown). These immunochemical results are consistent with previous findings (Ige et al., 2000; Charles et al., 2001). Thus, our control experiments indicated that the antibody against the GABA_B_R1 subunit was effective and specific.

### Data analysis

#### Analyses of western blotting results

For each case, levels of the GABA_B_R1 subunit, the GABA_B_R2 subunit, Actin, and α-Tubulin were evaluated by using gel images probed by respective primary antibodies. For each gel image, an optic density (OD) value was measured for each of the four bands corresponding to the ICc, ICd, ICx, and cerebellum. For each structure, the OD value for a receptor subunit (i.e., either the GABA_B_R1 or GABA_B_R2 subunit) was normalized against the OD value for α-Tubulin. A ratio was obtained between the normalized OD value of a collicular subdivision and the normalized OD value of the cerebellum. The ratios from all the cases studied were then used to obtain a mean and a standard error to reflect the level of a receptor subunit in a collicular subdivision in reference to that in the cerebellum.

#### Analyses of immunohistochemical images

Digital photomicrographic images were taken for each section probed by an antibody against the GABA_B_R1 or the GABA_B_R2 subunit. A grid with 150 × 150 μm squares (named as grid boxes elsewhere in the text) and an arbitrary origin was placed over the area of the IC (bottom panel of Figure 2B). The origin was used as a reference point for superimposing the outline of the IC and a contour showing the regional distribution of OD or cell body morphological characteristic in the IC (see below). Images were taken for all the grid boxes or alternating grid boxes in the IC using a 63X oil immersion objective at a focal plane 10 μm below the top surface of the tissue. These images were used to examine the number of GABA_B_R1- or GABA_B_R2-immunoreactive (GABA_B_R1-IR or GABA_B_R2-IR) cell bodies as well as the level of immunoreactivity and morphological features of these cell bodies. The images were also used to evaluate the neuropil level of subunit expression as well as the overall level (i.e., combined cell body and neuropil level) of expression. Images for each set of tissue samples were taken in multiple sessions. At the beginning of each imaging session, a predetermined area of a cerebellar section was examined and the mean OD of this area was measured (see below for measurement of OD) to ensure that illumination conditions of the microscope were consistent across different imaging sessions.

To assess the overall level of immunoreactivity in an area (e.g., an 150 × 150 μm grid box), a gray level was measured at each pixel within the area. In such a measurement, white and black colors corresponded to pixel values of 0 and 255, respectively. The mean and the standard deviation of all the pixel gray levels in the box were obtained to indicate the OD of this area. To assess the level of neuropil labeling within a grid box, five small square areas with 10 × 10 μm dimensions were randomly picked within the grid box. These squares were devoid of any cell bodies or parts of cell bodies. A gray level was measured at each pixel in these five areas and the mean and standard deviation of all the pixel gray levels were obtained to represent the OD of the neuropil in the grid box.

A normalized OD value was calculated for an area of interest by using the mean OD from the molecular layer of the cerebellum and the mean OD from an area with the lightest labeling in the entire set of section (typically white matter of the cerebellum):

$$Normalized\;OD=(OD_{aud}-OD_{l})/(OD_{cm}-OD_{l})$$

where *OD*_*aud*_ is the mean OD value of an area of interest (i.e., a grid box, five 10 × 10 μ m squares, or a cell body). *OD*_*cm*_ and *OD*_*l*_ are the mean OD values of the cerebellar molecular layer and the area with the lightest labeling, respectively.

Immunoreactive cell bodies were counted in each grid box. An immunoreactive cell body had an identifiable nucleus and a mean OD value higher than a threshold OD level for the grid box. This threshold level equaled the mean neuropil OD plus one standard deviation of the neuropil OD in the grid box. Only cell bodies with a major axis (i.e., longest axis) longer than 6 μm were counted.

For each labeled cell body, the level of labeling and the size of the cell body were examined. A cell body was outlined manually and the level of labeling in the cell body was obtained by calculating the mean OD within the outlined area. The perimeter, occupied area, length of the major axis, and orientation of the major axis were also measured for the cell body. The area occupied by a cell (*a*) and the length of the major axis (*l*) were used to calculate an elongation index (*EI*) to describe the shape of an immunoreactive cell body:

$$EI=1-\frac{a}{\frac{\pi }{4}l^{2}}$$

An EI value is within the range between 0 and 1. An elongated cell body results in a large EI value, while a perfect circular cell body results in an EI value of 0.

Measurements of OD values and cell body morphological features from all grid boxes were combined to calculate mean values or to create histograms for the three subdivisions. For this purpose, a grid box divided by a border between two subdivisions was assigned to the subdivision that covered a larger part of the grid box.

Image J software (U.S. National Institute of Health, Bethesda, MD) was used in the counting and analyses of immunoreactive neurons, and the measurements of OD values. DeltaGraph software (RedRock software, Salt Lake City, UT) was used for plotting contours, histograms, and vector charts to show regional distributions of immunoreactivity and morphological features of immunoreactive cell bodies. For the purpose of illustration, brightness and contrast of photomicrographic images was adjusted using Photoshop CS4 Extended software (Adobe Systems, San Jose, CA). Outlines of neural structures in photomicrographic images were traced using Illustrator (Adobe Systems, San Jose, CA). Areas outside the IC in a contour plot made using DeltaGraph were cropped using Illustrator.

## Results

### Levels of the GABA_B_R1 and GABA_B_R2 subunits in the IC: western blotting

Western blotting experiments were conducted using four rats. In three of the four animals, both the GABA_B_R1 and the GABA_B_R2 subunits were probed. In the fourth animal, only the GABA_B_R2 subunit was probed. As shown in Figure 1B, both the GABA_B_R1 and GABA_B_R2 subunits were expressed at a higher level in the ICd than in the other two collicular subdivisions. This area difference was confirmed by all the other cases examined in this study (2 and 3 cases for the GABA_B_R1 and GABA_B_R2 subunits, respectively). The GABA_B_R1/α-Tubulin ratio in the ICc and ICx were about 65 and 70% of that in the ICd, respectively. The GABA_B_R2/α-Tubulin ratio in the ICc and ICx were about 55 and 75% of that in the ICd, respectively.

### Localization of the GABA_B_R1 and GABA_B_R2 subunits: immunohistochemistry

Immunohistochemical experiments were conducted using a total of seven rats. In one of these rats, both the GABA_B_R1 and the GABA_B_R2 subunits were probed. In three rats the GABA_B_R1 subunit was probed, while in another three rats the GABA_B_R2 subunit was probed. Regional and cellular distributions of immunoreactivity were examined in each of these cases. For each subunit, OD values were measured in four cases to evaluate the distribution of neuropil and overall (combined cell body and neuropil) immunoreactivities over the entire IC. In three of the four cases OD values were measured in alternating grid boxes in each section, while in one of the four cases OD values were measured in all the grid boxes in each section. Comparisons made in the cases in which ODs were measured for all the grid boxes (one for each subunit) revealed that contours showing distributions of neuropil and overall immunoreactivity based on measurements from all the grid boxes were similar to those based on measurements from alternating boxes. Therefore, results presented in the following section will be based on measurements from alternating grid boxes for all cases in order to keep consistency. The level of cell body immunoreactivity, the density of labeled cell bodies, and the size and orientation of labeled cell body were examined in two cases for each subunit.

**Figure 3:**
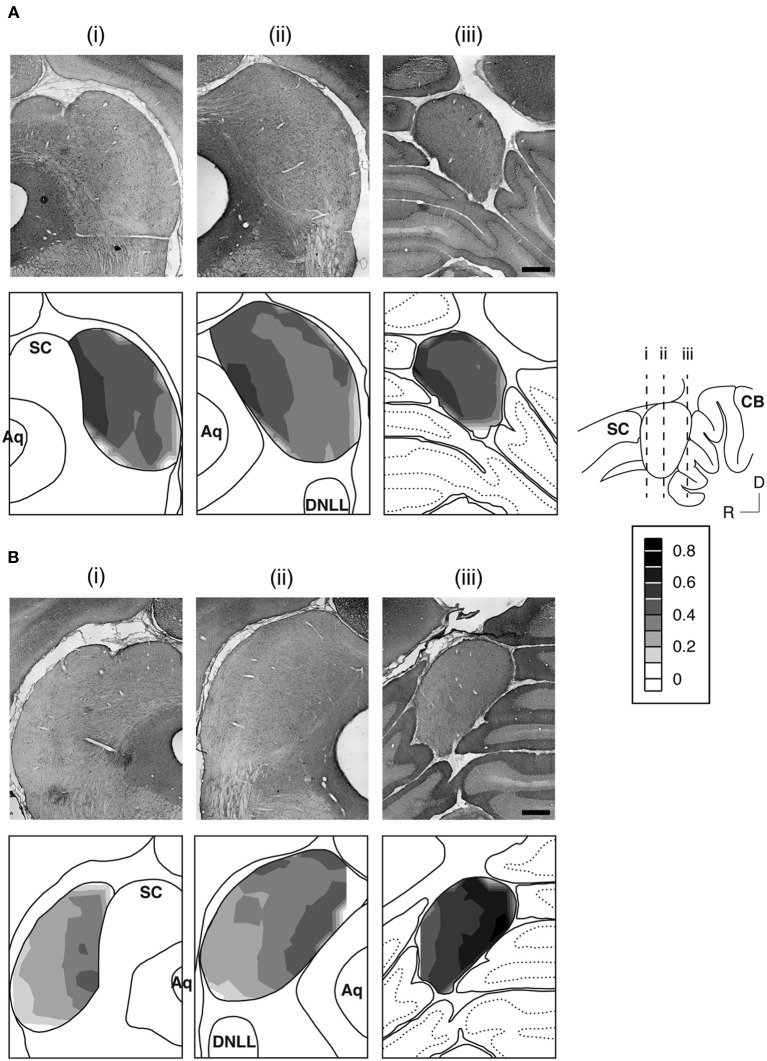
**An example showing immunoreactivity to antibodies against the GABA_B_R1 subunit (A) and the GABA_B_R2 subunit (B) at different rostrocaudaul locations of the IC**. The rostrocaudal locations of the sections shown in **(A)** and **(B)** are indicated in a sagittal diagram in the inset. Below each photomicrograph in **(A)** and **(B)** is a diagram of the section with a contour plot showing the distribution of a combined level of cell body and neuropil immunoreactivity over the area of the IC. For making a contour, a normalized OD was obtained for each of the alternating grid boxes over the area of the IC. Results in **(A)** and **(B)** are from a single animal. Scale bars in **(A)** and **(B)**: 500 μm.

The GABA_B_R1 and GABA_B_R2 subunits were found throughout the entire IC (Figures 2B and 3). For a section from the middle portion of the rostrocaudal extent of the IC [Figures 3A(ii),B(ii)], the overall level of expression was higher in the dorsomedial region (i.e., the ICd) than the ventrolateral region of the IC (i.e., the ventral parts of the ICx and ICc). The dorsal parts of the ICc and ICx also had relatively high level of expression. The reduction in the level of immunoreactivity from dorsomedial to the ventrolateral part of the IC was gradual. No defined boundaries were found among the three subdivisions. In the rostral part of the IC, an area defined as the rostral cortex of the IC by recent publications (Loftus et al., 2008; Malmierca et al., 2011), the level of immnoreactivity was higher in the medial than in the lateral region [Figures 3A(i),B(i)]. Stronger labeling in the medial than the lateral region of the IC was also observed in the caudal part of the IC [Figures 3A(iii),B(iii)]. Across the rostrocaudal extent of the IC, the level of labeling appeared to be higher in the caudal than the rostral part. This rostrocaudal difference is more apparent for GABA_B_R2 immunoreactivity than GABA_B_R1 immunoreactivity.

Over the area of the IC in a section, a mean OD value was obtained for each of the alternating grid boxes to reflect the combined level of cell body and neuropil labeling in the boxes. A contour plot was created by using the mean OD values from these grid boxes. Contour plots for sections shown in Figure 3 indicate that the GABA_B_R1 and GABA_B_R2 subunits displayed similar distributions of a combined level of cell body and neuropil immunoreactivity. For both subunits, a high level of immunoreactivity was observed in the medial/dorsomedial part of the IC. Measurements of OD values in three additional cases for each subunit supported findings from the case shown in Figure 3.

**Figure 4:**
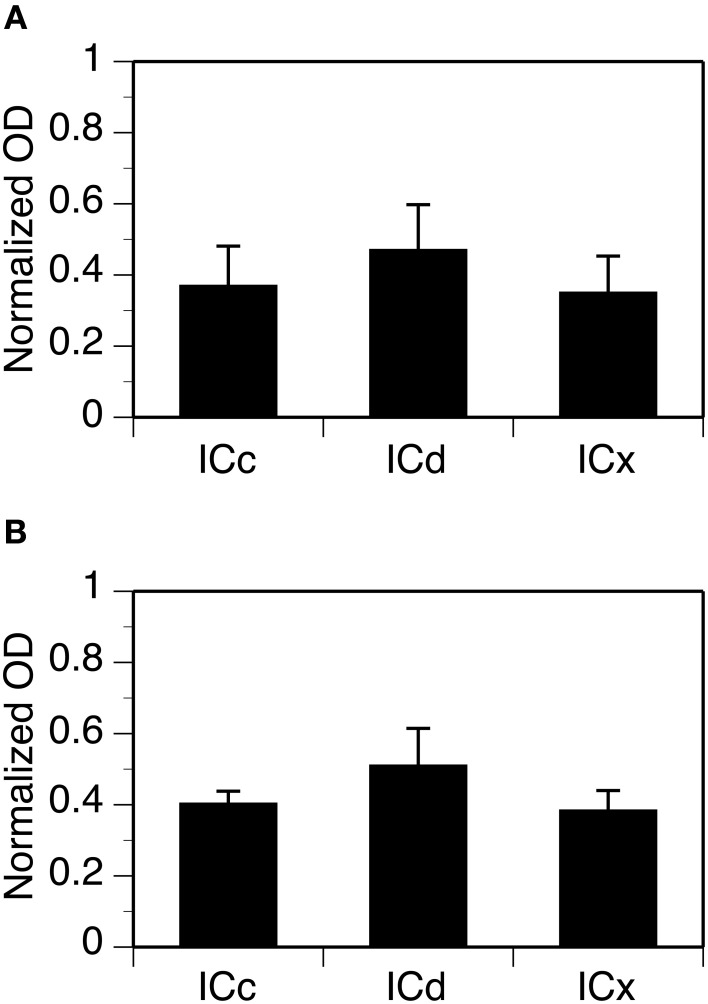
**Group results from immunohistochemical experiments showing overall (cell body and neuropil combined) levels of GABA_B_R1 and GABA_B_R2 immunoreactivity (A and B) in three subdivisions of the IC**. For each section in an animal, a normalized OD was obtained for each of the alternating grid boxes. A mean normalized OD value was obtained for each collicular subdivision of an animal. Each bar in a bar chart represents a grand mean value for four animals for a subdivision. Error bars indicate standard errors.

For each case, OD values from alternating grid boxes in an entire set of sections were combined to generate three mean OD values to reflect overall levels of GABA_B_R1 or GABA_B_R2 immunoreactivity in the ICc, ICd, and ICx. Group results from four cases for each subunit confirmed that the overall levels of GABA_B_R1 and GABA_B_R2 immunoreactivity were higher in the ICd than in the ICc and the ICx (Figure 4). For both subunits, levels of immunoreactivity in the ICc and ICx were about 75% of that in the ICd.

**Figure 5:**
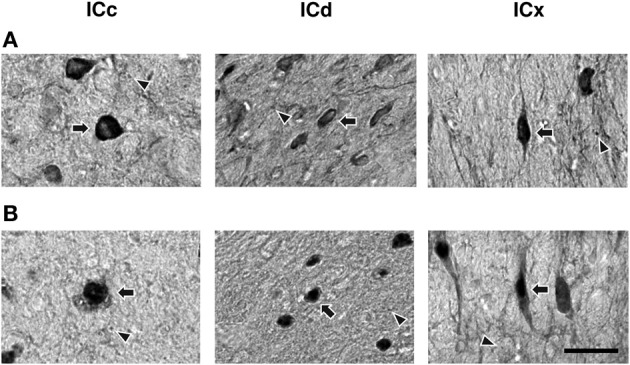
**High magnification photomicrographs showing GABA_B_R1 and GABA_B_R2 immunoreactivity (A and B) in the ICc, ICd, and ICx**. Arrows point toward labeled cell bodies while arrowheads point toward labeled puncta in the neuropil. Scale bars: 25 μm.

Higher magnification images revealed that labeling of cell bodies in the IC by an antibody against the GABA_B_R1 or GABA_B_R2 subunit was either punctate or diffused (Figure 5). Punctate labeling was observed in the neuropil of the IC. Cell bodies immunoreactive to the GABA_B_R1 antibody typically had strong labeling on or close to the cell membrane, while those immunoreactive to the GABA_B_R2 antibody typically had strong labeling throughout the cell body including areas in and close to the nucleus.

**Figure 6:**
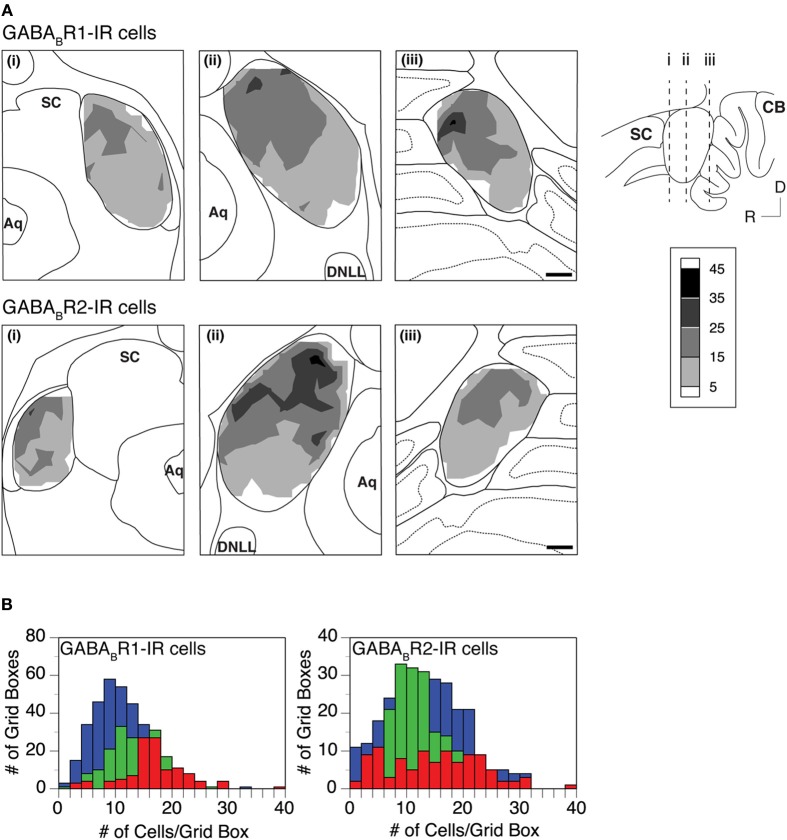
**(A)** Densities of GABA_B_R1-IR and GABA_B_R2-IR cells (top and bottom rows) in the IC. Analyses were conducted on two animals for each subunit. Results in the two rows are from two different animals. In each row, contour plots in three panels show densities of labeled cells in three coronal sections at different rostrocaudal locations as indicated in the inset. A density of labeled cells represents the total number of immunoreactive cells in a 150 × 150 μm grid box. Each contour is made by using densities of cells in alternating grid boxes over the area of the IC. **(B)** Histograms showing distributions of GABA_B_R1-IR cells (left panel, results from the same animal as in the top row in **A**) and GABA_B_R2-IR cells (right panel, results from the same animal as in the bottom row in **A**) in the ICc (green), ICd (red), and ICx (blue). Scale bars in **(B)**: 500 μm.

Distributions of GABA_B_R1-IR and GABA_B_R2-IR cell bodies in the IC were quantitatively examined in two cases for each subunit. For each case, the density of immunoreactive cell bodies was examined in an entire set of tissue sections. In each section, immunoreactive cell bodies were counted in all the alternating grid boxes. As shown by an example in Figure 6, labeled cell bodies were densely packed in the dorsomedial region of the IC in sections from the mid portion of the rostrocaudal extent of the structure [Figure 6A(ii) top and bottom panels]. Cell packing density was low in the ventral region. In the rostral part of the IC, the density of labeled cells appeared to be slightly higher in the dorsal or dorsolateral than the other regions [Figure 6A(i) top and bottom panels]. In sections close to the caudal pole of the IC, density of labeled cells was higher in the dorsal than the ventral region [Figure 6A(iii) top and bottom panels]. The overall density of labeled cell bodies was reduced at the caudal pole, with the dorsal-ventral contrast still observed (data not shown). Densities of labeled cell bodies in all of the alternating grid boxes in the entire set of sections were summarized in histograms shown in Figure 6B. Distributions of the density of immunoreactive cell bodies peaked at higher values in the ICd than in the ICc and ICx for the GABA_B_R1 subunit (Figure 6B left panel). The mean number of labeled cells/grid box supported that GABA_B_R1-IR cells were more densely packed in the ICd than in the ICc and ICx (Table 1). For the GABA_B_R2 subunit, the difference in the distribution of density of immunoreactive cell bodies among the three subdivisions was smaller than for the GABA_B_R1 subunit (Figure 6B right panel). However, the ICd still had the highest average density of labeled cells among the three collicular subdivisions (Table 1). The relatively small difference was likely partially related to the fact that few GABA_B_R2-IR cells were found in the caudal pole of the ICd. The area difference in the packing density of immunoreactive cells was confirmed by quantitative analysis of results from a second case for each subunit.

**Table 1:** **Summary of results from two animals for GABA_B_R1-IR and GABA_B_R2-IR cell bodies, respectively**.

	**GABA_B_R1-IR cells mean ± SD**			**GABA_B_R2-IR cells mean ± SD**		
	**ICc**	**ICd**	**ICx**	**ICc**	**ICd**	**ICx**
Total number of neurons examined	2538	1879	3476	2038	1380	3508
Number of cells/grid box	13.4 ± 5.0	16.3 ± 5.7	10.4 ± 4.9	12.0 ± 5.1	14.5 ± 8.2	12.9 ± 6.8
Normalized optic density	0.77 ± 0.12	0.76 ± 0.12	0.76 ± 0.13	0.98 ± 0.17	1.06 ± 0.16	0.98 ± 0.17
Area of cell body (μm^2^)	67.0 ± 34.6	58.0 ± 26.5	63.4 ± 35.2	69.1 ± 45.9	53.8 ± 27.8	66.7 ± 42.0
Perimeter of cell body (μm)	32.3 ± 11.1	30.3 ± 9.6	31.9 ± 12.0	32.4 ± 12.3	28.7 ± 9.4	32.4 ± 12.1
Major axis of cell body (μm)	11.5 ± 4.1	11.0 ± 3.7	11.6 ± 4.5	11.4 ± 5.0	10.3 ± 3.5	11.7 ± 4.5

**Figure 7:**
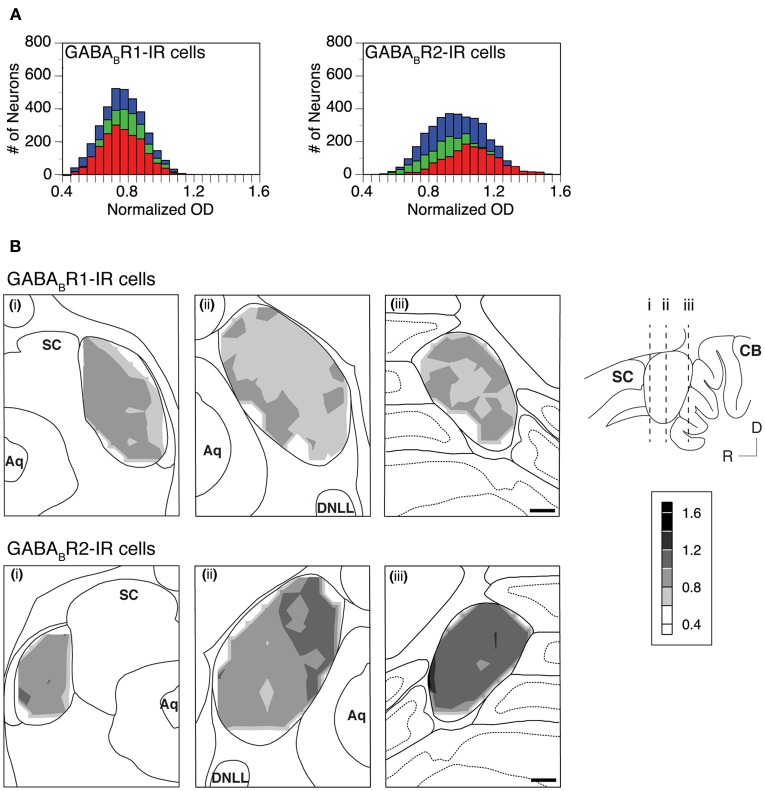
**(A)** Histograms showing distributions of the normalized OD for individual GABA_B_R1-IR cell bodies (left panel) and GABA_B_R2-IR cell bodies (right panel) in the ICc (green), ICd (red), and ICx (blue). Analyses were conducted on two animals for each subunit. Results in the left and right panels are from two different animals. **(B)** Regional distribution of the mean normalized OD of GABA_B_R1-IR cells (top panels) and GABA_B_R2-IR cells (bottom panels) over the area of the IC in three coronal sections at different rostrocaudal locations as indicated in the inset. The mean normalized OD represents the mean of the normalized ODs of all the labeled cells in a 150 × 150 μm grid box. A contour is made by using the mean normalized OD values from alternating grid boxes over the area of the IC. Scale bars in **(B)**: 500 μm.

The level of cell body immunoreactivity was examined in two cases for each subunit. For each case, ODs of immunoreactive cell bodies were measured using an entire set of tissue sections and measurements were conducted in each of the alternating grid boxes in each section. Results shown in Figure 7A revealed a normal distribution of cell body OD in each of the three collicular subdivisions. For the GABA_B_R1 subunit, the distributions of OD peaked at a value about one fourth lower than the mean OD of the cerebellar molecular layer (Figure 7A left panel). For the GABA_B_R2 subunit, the distributions of the cell body OD in the ICc and ICx peaked at a level slightly lower than the mean OD of the cerebellar molecular layer (Figure 7A right panel). In contrast, the distribution in the ICd peaked at a value slightly higher than the mean OD of the cerebellar molecular layer. The similarity in the level of cell body GABA_B_R1 immunoreactivity among the three subdivisions and the small difference in the cell body GABA_B_R2 immunoreactivity between the ICd and ICc/ICx were also reflected by mean OD values shown in Table 1.

A mean value was obtained for the OD values of all the immunoreactive cell bodies in each of the alternating grid boxes in a section. These mean OD values were used to create a contour to show the regional distribution of cell body immunoreactivity. Contour plots in Figure 7B revealed small variations in the mean OD over the IC. These variations resulted in a patchy pattern over the IC for GABA_B_R1 immunoreactivity (Figure 7B top panels). For GABA_B_R2 immunoreactivity, OD values were slightly higher in sections from the caudal part of the IC and the dorsomedial region of the IC in sections from the mid rostrocaudal extent of the structure (Figure 7B bottom mid and right panels). Relatively high OD values in these regions were in agreement with the fact that individual GABA_B_R2-IR cell body ODs had a distribution that peaked at a higher value in the ICd than in other collicular subdivisions (Figure 7A right panel). The regional distribution of cell body OD shown in Figure 7 was verified by one additional case for each subunit.

**Figure 8:**
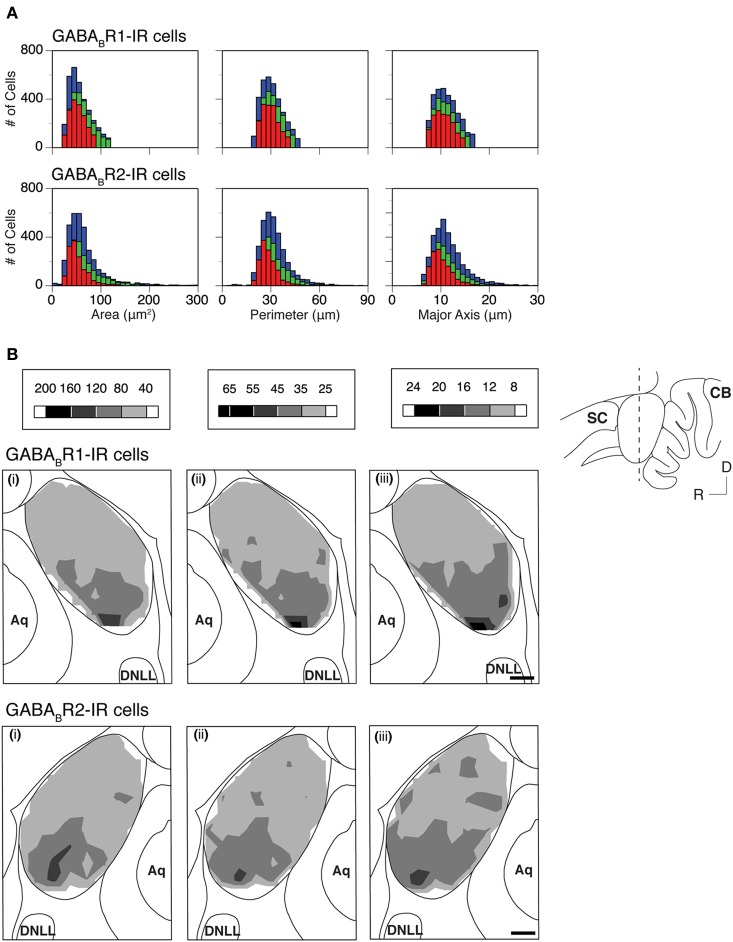
**(A)** Histograms showing distributions of the area (left panels), perimeter (middle panels), and major axis (right panels) for individual GABA_B_R1-IR cell bodies (top panels) and GABA_B_R2-IR cell bodies (bottom panels) in the ICc (green), ICd (red), and ICx (blue). Analyses were conducted on two animals for each subunit. Results in the top and bottom panels are from two different animals. **(B)** Regional distributions of the mean area **(i)**, mean perimeter **(ii)**, and mean major axis **(iii)** of GABA_B_R1-IR cells (top row) and GABA_B_R2-IR cells (bottom row) over the IC in a coronal section as indicated in the inset. A mean area, a mean perimeter, and a mean major axis were obtained by using the corresponding parameters from all the labeled cells in a 150 × 150 μm grid box. A contour is made by using the mean values from all the alternating grid boxes over the area of the IC. Results in the top panels of **(B)** are from the same animal as in the top panels of **(A)** while results in the bottom panels of **(B)** are from the same animal as in the bottom panels of **(A)**. Scale bars in **(B)**: 500 μm.

The area, perimeter, and major axis of an immunoreactive cell body were measured over the IC in two cases for each subunit. For each case, measurements were made in alternating grid boxes in each section. Histograms were made to show distributions of cell body area, perimeter, and major axis in the ICc, ICd, and ICx. Results from the two cases shown in Figure 8A indicated that GABA_B_R1-IR and GABA_B_R2-IR cell bodies tended to have smaller sizes in the ICd than in the ICc and ICx. For both cases, grand mean values for cell body size parameters in the three subdivisions supported that immunoreactive cell bodies tended to have smaller sizes in the ICd (Table 1).

Mean area, perimeter, and longest axis were obtained for all immunoreactive cell bodies in a grid box for the two cases shown in Figure 8A. Contour plots based on these mean values revealed that at the mid portion of the rostrocaudal extent of the IC, immunoreactive cell bodies tended to be smaller in the dorsomedial region (ICd) than in the ventral region (ventral ICc and ICx) (Figure 8B). In rostral and caudal part of the IC, neurons in the dorsal region also tended to have relatively small cell body sizes. However regional differences seemed smaller (data not shown). The area differences in cell body size as shown in Figure 8 were verified by one additional case for each subunit.

**Figure 9:**
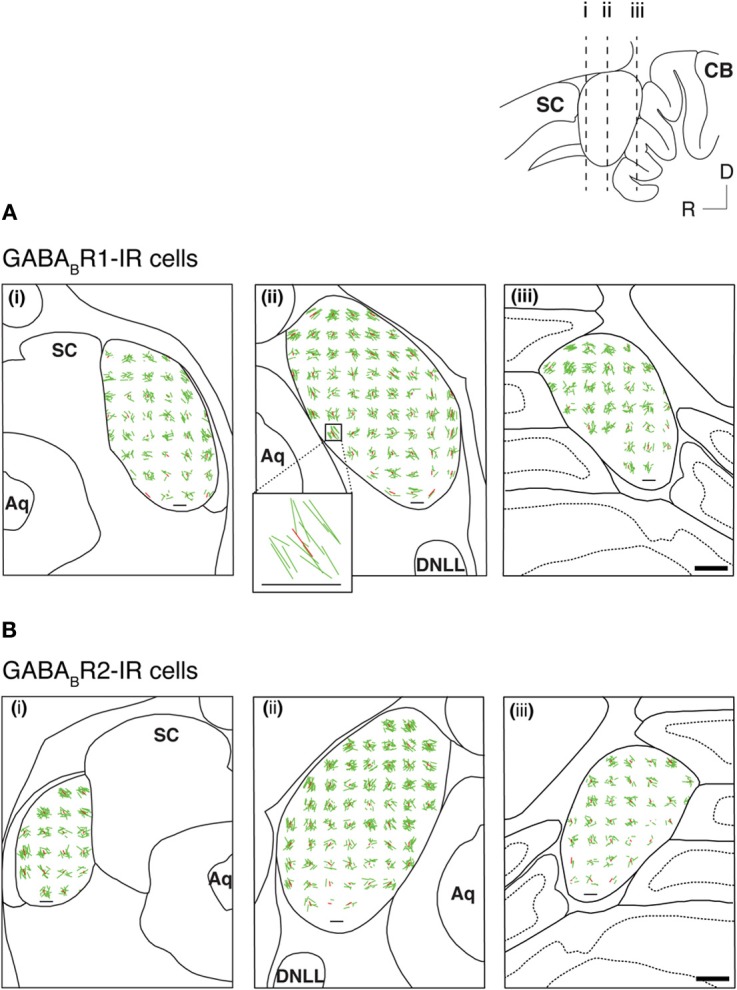
**Vector plots reflecting shapes and orientations of GABA_B_R1-IR cell bodies (A) and GABA_B_R2-IR cell bodies (B) over the IC in three coronal sections at different rostrocaudal levels as indicated in the inset**. Analyses were conducted on two animals for each subunit. Results in **(A)** and **(B)** are from two different animals. In each section, shapes and orientations of labeled cell bodies were measured in alternating 150 × 150 μ m grid boxes. Each green line is a vector representing results from one individual neuron, with the length indicating the elongation index (EI) and the orientation indicating the orientation of the major axis of the neuron. Each red line indicates the mean of individual vectors in a grid box. Inset shows individual vectors and the mean vector in a grid box located at the ventromedial region of the IC in a section from the middle rostrocaudal level of the IC of the case shown in **(A)**. A small horizontal black line in the area of IC in each panel and the horizontal line at the bottom of the inset indicate an EI value of 1. Scale bar (thick horizontal line) in **A(iii)** and **B(iii)**: 500 μm.

Many GABA_B_R1-IR and GABA_B_R2-IR neurons in the IC display elongated cell bodies (see Figure 5 for examples). The elongated shape of a cell body was quantitatively described by using an EI (see section “Experimental Procedures” for details). For each section, an EI was calculated for each of the cell bodies in all the alternating grid boxes over the area of the IC. A vector plot was made by using EI values and angles of the major axes of all these cell bodies. Figure 9 displays results from two cases respectively for the GABA_B_R1 and GABA_B_R2 subunits. For sections obtained from the mid portion of the rostrocaudal extent of the IC [Figures 9A(ii),B(ii)], vectors close to the outline of the IC had a tendency of orientation parallel to the outline. In contrast, vectors in the ICc region did not show a strong tendency of orientation. A mean vector was calculated for each grid box by using the EIs and the angles of major axes of individual neurons. Mean vectors in grid boxes along the outline of the IC were larger and had a tendency to follow the outline. In contrast, mean vectors in the ICc region were small and did not show a tendency of orientation. These results are consistent with observations from individual neurons. In the rostral and caudal parts of the IC, no strong tendency of orientation was found, although many neurons in these parts display elongated shapes [Figures 9A(i),(iii),B(i),(iii)].

**Figure 10:**
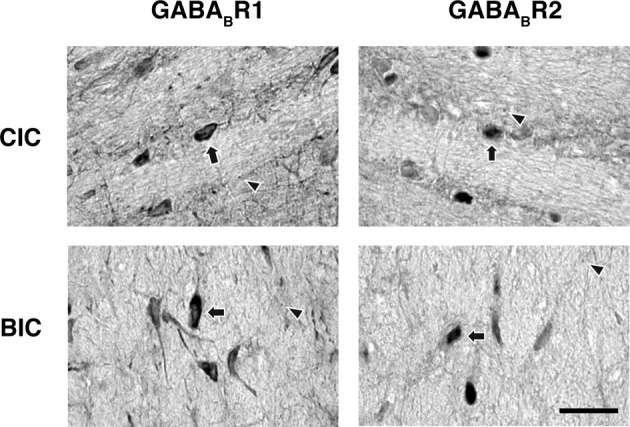
**Photomicrographs showing GABA_B_R1 and GABA_B_R2 immunoreactivity in the CIC and BIC regions**. Arrows point toward labeled cell bodies while arrowheads point toward labeled puncta in the neuropil. Scale bars: 25 μm.

Neurons immunoreactive to the GABA_B_R1 and GABA_B_R2 antibodies were found among fibers in the commissure of the inferior colliculus (CIC) and the brachium of the inferior colliculus (BIC) (Figure 10). In the CIC, immunoreactive cell bodies existed over the entire lateral-medial extent. These cell bodies had elongated shapes (as indicated by arrows), which were oriented in parallel with the commissural fibers. The level of labeling of these cell bodies was similar to that of cell bodies in the ICd. Immunoreactive cell bodies in the BIC also had elongated shapes. These cells tended to have a vertical orientation and the level of labeling of these cells was similar to that of cell bodies in the ICx.

**Figure 11:**
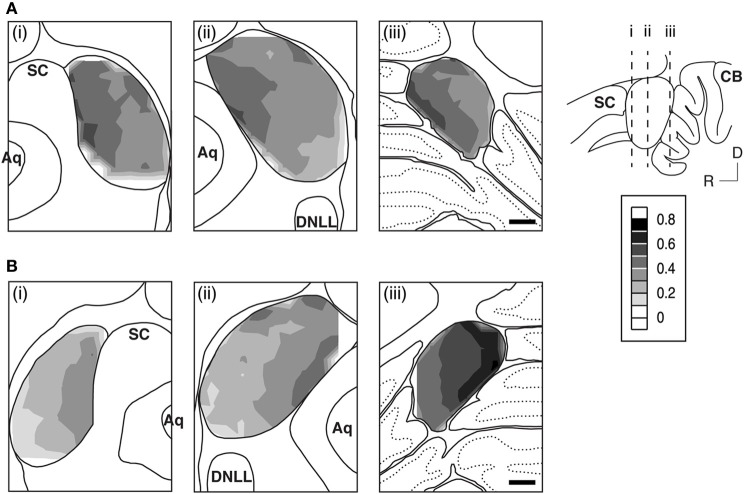
**An example showing regional distributions of the level of neuropil immunoreactivity to antibodies against the GABA_B_R1 (A) and the GABA_B_R2 (B) subunits over the IC in sections from different rostrocaudaul locations of the IC indicated in the inset**. For making a contour, normalized OD values were obtained for the neuropil in each of the alternating grid boxes over the area of the IC. Results in **(A)** and **(B)** and those in Figure 3 are from the same animal. Scale bars in **(A)** and **(B)**: 500 μm.

**Figure 12:**
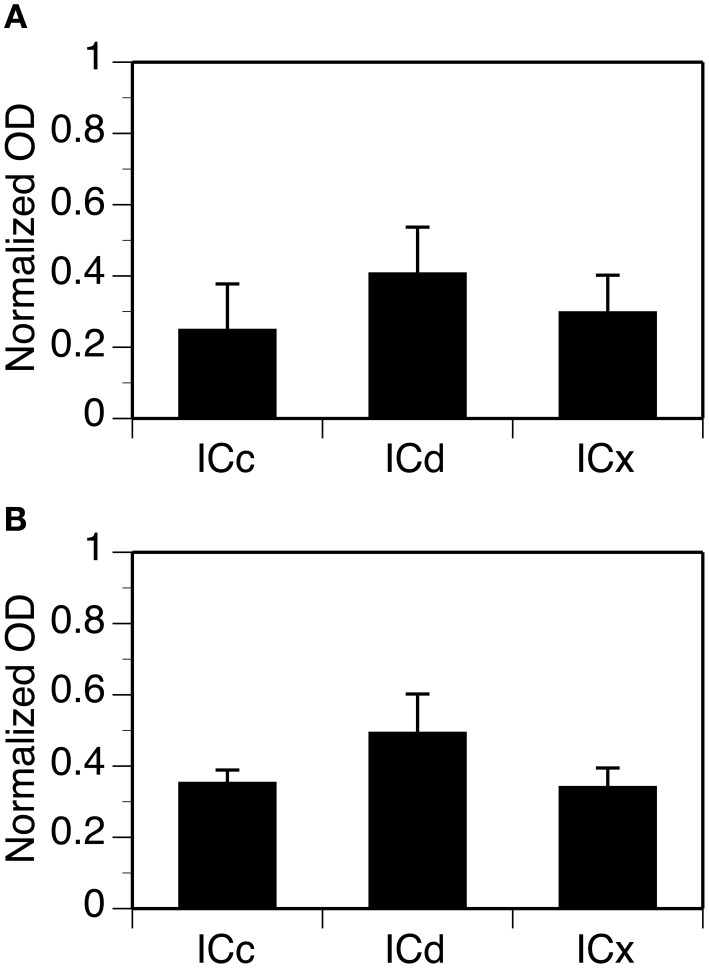
**Group results showing levels of GABA_B_R1 (A) and GABA_B_R2 (B) immunoreactivity in the neuropil in three subdivisions of the IC**. For each section in an animal, normalized OD was obtained for each of the alternating grid boxes. A mean normalized OD value was obtained for each collicular subdivision. Each bar in a bar chart represents a grand mean value for four animals for a subdivision. Error bars indicate standard errors.

Neuropil OD values were measured in four cases for each subunit. In each case, measurements were conducted in alternating grid boxes in each section (see “Experimental Procedures”) and a contour plot was made for the section. Results indicated that immunoreactivity for both the GABA_B_R1 and the GABA_B_R2 subunits was higher in the medial/dorsomedial part than the ventrolateral part of the structure (Figures 11A,B). For each case, OD values from alternating grid boxes over the area of IC in the entire set of sections were combined and three grand mean values were obtained for the three collicular subdivisions. Group results (four cases for each subunit) indicated that the ICd had a higher level of neuropil labeling than the ICc and ICx (Figure 12).

## Discussion

### Technical considerations

Sections at 30 μm thickness were used in our immunohistochemical experiments. Strengths of immunoreaction might not have been even at different depths of a section. In an effort to minimize this effect, all photomicroscopic images were taken when the focal plane was at a depth of 10 μm below the tissue surface. In spite of this effort, differences might have existed in immunopenetration between sections treated with antibodies against the GABA_B_R1 and GABA_B_R2 subunits, respectively. These differences might have, to some extent, introduced disparities between the two subunits in the distribution of immunoreactivity.

The relatively thick tissue section might have also affected measurements of OD values. Although measured OD values were mainly determined by photons from the focal plane, contributions from off-focal planes should not be completely ignored. Photons from off-focal planes would not necessarily affect the evaluation of the distribution of the overall level of immunoractivity. It might have, to some extent, smeared distributions of neuropil and cell body immunoreactivity, as measurements of cell body labeling in the focal plane could be affected by neuropil in the off-focal planes and vice versa. Even with this possible effect, a difference between the neuropil and cell bodies in the area distribution of immunoreactivity was evident (Compare Figures 7 and 11).

### Level and distribution of the GABA_B_R1 and GABA_B_R2 subunits over the IC

Our immunohistochemical and Western blotting experiments reveal that the combined level of cell body and neuropil expression is higher in the ICd than in the ICc and ICx for both GABA_B_R1 and GABA_B_R2 subunits. Results based on the two techniques revealed that for both subunits, the combined level of expression in the ICc and ICx was about two thirds of that in the ICd.

Results from the present study support our previous qualitative observation regarding the distribution of the GABA_B_R2 subunit in the IC (Jamal et al., 2011). The results also support findings from receptor autoradiographical studies indicating that functional GABA_B_ receptors are expressed at a higher level in the dorsomedial region of the IC and a lower level in the ventral region of the structure (Big brown bat: Fubara et al., 1996; Rhesus monkey: Hilbig et al., 2007; Rat: Bowery et al., 1987; Milbrandt et al., 1994). Results from the rhesus monkey revealed that the receptor had a higher level in the rostral than the caudal part of the ICd (Hilbig et al., 2007). This difference was not observed in the present study.

A question arises as to whether the area differences in the level of combined cell body and neuropil level of immunoreactivity are mainly dependent on the immunoreactivity in cell bodies, the neuropil, or both. For each of the two subunits, the neuropil OD value was higher in the ICd than the other regions of the IC while the cell body OD had very small regional differences over the IC. A relatively high neuropil OD value in the ICd certainly contributed to the high overall level of labeling in this region as the brain tissue in the IC was predominately occupied by the neuropil. The contribution of immunoreactive cell bodies to the overall level of labeling cannot be compared by only using cell body OD values. Results shown in Figures 6 and 8 suggest that the combined area of cell bodies in a grid box in the ICd was almost equal to or slightly larger than that in the ICc and ICx. Along with distribution of cell body OD over the IC, the slightly larger combined cell body area in the ICd likely resulted in a slightly larger contribution of cell bodies to the overall level of labeling in the ICd.

Regional dependences in the level of GABA_B_R1 and GABA_B_R2 did not result in clear boundaries within the IC. Contour plots based on cell body, neuropil, and combined cell body and neuropil OD values had shapes different from the borders between different collicular subdivisions (Paxinos and Watson, 2007; Loftus et al., 2008; Malmierca et al., 2011). Differences in the level of GABA_B_R1 and GABA_B_R2 immunoreactivity also existed among different regions within a collicular subdivision. The differences were especially apparent in the ICc and ICx. Within each of these two subdivisions, levels of immunoreactivity were higher in the dorsal than the ventral region. Therefore, it is not appropriate to use the level of expression of the GABA_B_ receptor to define borders of IC subdivisions.

The GABA_B_R1 and GABA_B_R2 subunits had similar regional distributions over the IC. This similarity is consistent with findings from many other brain structures (White et al., 1998; Kuner et al., 1999; Ige et al., 2000; Charles et al., 2001; López-Bendito et al., 2002; Kulik et al., 2003; Panzanelli et al., 2004; Marshall and Foord, 2010). It supports that a functional GABA_B_ receptor is a heterodimer consisting of both subunits.

In spite of the similarity in regional distribution, differences were observed between the two subunits in cellular/subcellular distribution. For example, a GABA_B_R2-IR neuron typically had strong labeling throughout the cell body. In contrast, a GABA_B_R1-IR neuron typically had stronger labeling on and close to the cell membrane than the rest of the cell body. Similar differences were found by previous studies in other brain structures (Charles et al., 2001; Panzanelli et al., 2004). In the present study, both subunits were visualized using DAB reaction product. Therefore, cellular/subcellular distributions of labeling cannot be directly used to localize functional GABA_B_ receptors in individual IC neurons. Colocalization of the two subunits and distribution of functional GABA_B_ receptors can be studied only by conducting double labeling experiments involving two different fluorophores for visualizing two subunits respectively. As shown by previous studies, GABA_B_R1 and GABA_B_R2 subunits are made separately in the endoplasmic reticulum before trafficking to the cell membrane to form functional receptors (Pin et al., 2004; Restituito et al., 2005; Pooler and McIlhinney, 2007) or to the cell nucleus to regulate gene expression (Gonchar et al., 2001). Immunoreactivity against a subunit observed in the present study not only reflected the subunit molecules in functional receptors but also those available for making functional receptors. The disparity between the cellular/subcellular distributions of GABA_B_R1 and GABA_B_R2 immunoreactivity very likely indicates that a difference exists between the two subunits in the distribution of available molecules for making functional receptors.

For both the GABA_B_R1 and the GABA_B_R2 subunits, labeled puncta were found on cell bodies as well as in the neuropil. In addition, diffused cytoplasmic labeling was found in cell bodies. While labeled puncta on cell bodies likely indicated the sites where post- and/or presynaptic GABA_B_ receptors were located, those in the neuropil could be either associated with bouton synapses or cross-sections of small dendrites or axons. An ultrastructural examination has to be conducted to determine whether the puncta in the neuropil represent functional GABA_B_ receptors. Diffused labeling in cell bodies likely reflected the subunits available for making functional receptors, although the possibility that it was directly associated with functional receptors could not be ruled out.

Previous anatomical studies have found that neurons in the IC have specific orientation and thickness of dendritic arbors (Oliver and Morest, 1984; Faye-Lund and Osen, 1985; Malmierca et al., 1995, 2011; Oliver, 2005). Disc-shaped (or flat) and stellate (or less-flat) cells are the two major cell types in the ICc. About 80% of the neurons in the ICc are disc shaped. Dendritic fields of disc-shaped cells are oriented with similar directions and fibrodendritic laminae are formed among these dendritic fields. Labeling of cell bodies along with proximal and secondary dendrites using HRP has revealed that most of the cell bodies of ICc neurons with disc-shaped dentritic fields have fusiform or oval shapes (Oliver, 1984). For these neurons, cell bodies have the same orientations as the dendritic fields, which are highly oriented in a ventrolateral to dorsomedial direction. With Nissl staining, although dendritic fields are not visible, a similarity in orientation among cell bodies is still evident (Oliver, 1984). In the present study, no strong tendency was observed in the orientations of immunoreactive cell bodies in the ICc. Further examinations are needed to find whether this lack of similar orientations indicates that most GABA_B_ receptor-immunoreactive cell bodies are outside fibrodendritic laminae in the ICc. Dendritic fields of neurons in the ICx and ICd are less oriented (Malmierca, 1991; Oliver et al., 1991; Malmierca et al., 2011). In the mid portion of the rostrocaudal extent of the IC, neurons in the deep layer of the ICx have dendritic fields perpendicular to the surface of the structure (Malmierca et al., 2011). Neurons located in the ICd bordering the ICc appear to have elongated dendritic fields and are oriented at an obtuse angle with the fibrodendritic laminae of the ICc (Malmierca et al., 2011). Our immunohistochemical results indicate that cell bodies in these regions tend to orient along the surface of the structure, suggesting that orientations of cell bodies and dendritic fields can be different in the ICd and ICx.

### Expression of GABA_B_ receptor subunits and synaptic inputs

The existence of the GABA_B_R1 and GABA_B_R2 subunits in the ICc is in agreement with inputs to this subdivision. Inputs to the ICc are predominantly from brainstem structures (see Cant, 2005; Schofield, 2005 for review). Some of these projections are GABAergic (Saint Marie et al., 1989; Saint Marie and Baker, 1990; Li and Kelly, 1992; Shneiderman et al., 1993; Oliver et al., 1994; Riquelme et al., 2001). Within the ICc, levels of immunoreactivity against the two subunits were relatively high in the dorsal than the ventral region. This difference is likely due to the differences in the input to these two regions. While the dorsal region of the ICc receives inhibitory projections predominantly from the dorsal nucleus of the lateral lemniscus, the ventral part of the ICc receives inhibitory projections predominantly from the superior olivary complex (Malmierca and Merchán, 2004). It has been shown that the dorsal nucleus of the lateral lemniscus is a major source of GABAergic projections (Shneiderman et al., 1988; Zhang et al., 1998; Chen et al., 1999). In contrast, the superior olivary complex is a major source of glycinergic projections, although GABAergic projections are also provided by this structure (Saint Marie et al., 1989; Saint Marie and Baker, 1990; Kulesza and Berrebi, 2000; Saldaña et al., 2009).

Cell bodies and the neuropil in the ICd and the dorsal part of the ICx are heavily labeled by antibodies against the two GABA_B_ receptor subunits. This observation is in contrast to the extrinsic inputs to these two collicular subdivisions. Major auditory inputs to these two subdivisions are from the auditory cortex (AC) and the medial geniculate nucleus (MGN) (Kuwabara and Zook, 2000; Winer, 2006). Projections from the AC are likely glutamatergic (Feliciano and Potashner, 1995). Projections from the MGN are also likely excitatory, as less than 1% of neurons in the rat's MGN are GABAergic (Winer and Larue, 1996). Therefore, GABA_B_ receptors in the ICd and ICx cannot be associated with direct inputs from the two forebrain structures. Possible sources of projections for activating GABA_B_ receptors in the ICd and ICx include local interneurons driven by descending inputs from the AC or the MG as well as neurons in the ICc. *In vivo* neurophysiological experiments revealed that electrical stimulation of the AC results in a delayed and long-lasting inhibitory effect on responses to sounds in the IC (Syka and Popelár, 1984; Torterolo et al., 1998; Bledsoe et al., 2003). These results support the existence of local GABAergic interneurons innervated by cortico-collicular projections.

The existence of GABA_B_R1 and GABA_B_R2 subunits in cell bodies and the neuropil in the CIC region is interesting. It is well known that the left and right inferior colliculi are connected by the CIC (Malmierca et al., 2009b). Some of the CIC fibers are GABAergic (Hernández et al., 2006). Therefore, the possibility exists that some GABA_B_ receptors in the IC are associated with CIC inputs. *In vitro* physiological recordings have indicated that inhibitory postsynaptic potentials elicited in IC neurons by stimulation of CIC fibers can be completely blocked by bicuculline, an antagonist for the GABA_A_ receptor (Smith, 1992; Moore et al., 1998). It is therefore unlikely that commissural stimulation can directly activate postsynaptic GABA_B_ receptors. Inhibitory postsynaptic potentials elicited by stimulation of CIC display paired-pulse inhibition that can be suppressed by an antagonist of the GABA_B_ receptor (Li et al., 1999). This finding suggests that stimulation of CIC can lead to the activation of presynaptic GABA_B_ receptors that regulate the release of inhibitory neurotransmitters. However, it is still unknown whether these GABA_B_ receptors are directly activated by CIC fibers or indirectly through local interneurons. Further research is needed for finding the relationship between GABA_B_ receptors and CIC projections.

### Area differences in receptor expression and synaptic functions

As revealed by the present study, area differences exist in the level and cellular distribution of the GABA_B_ receptor in the IC. While the level of cell body immunoreactivity was very similar in different collicular subdivisions, the level of neuropil immunoreactivity was higher in the ICd than in the other collicular subdivisions. Concomitantly, area differences exist in the synaptic function of the receptor in the IC. In the ICc, GABA_B_ receptors only exist at presynaptic sites and are responsible for the regulation of the release of glutamate and GABA (Hosomi et al., 1997; Lo et al., 1998; Ma et al., 2002; Sun et al., 2006). In the ICd, GABA_B_ receptors exist at both pre- and postsynaptic sites, with postsynaptic GABA_B_ receptors involved in the mediation of inhibitory postsynaptic potentials and presynaptic GABA_B_ receptors involved in the regulation of release of glutamate and GABA (Sun and Wu, 2009). Similar to results from other neural structures including the hippocampus and substantia nigra pars compact (Isaacson et al., 1993; Kulik et al., 2003; Saitoh et al., 2004), inhibitory postsynaptic currents mediated by GABA_B_ receptors in the ICd can only be evoked by electrical pulses presented at a high repetition rate (Sun and Wu, 2009). In many brain structures, inhibitory postsynaptic potentials mediated by GABA_B_ receptor at a high rate of stimulation are elicited as a result of spillover of GABA from the synaptic cleft as postsynaptic GABA_B_ receptors exist mainly at extrasynaptic sites of the cell membrane (Ige et al., 2000; Gonchar et al., 2001; Kulik et al., 2002, 2003). Our results regarding higher neuropil levels of the GABA_B_R1 and GABA_B_R2 in the ICd than ICc likely suggest that the postsynaptic function of the GABA_B_ receptor in the ICd is associated with axodendritic synapses in the neuropil. The presynaptic function of the GABA_B_ receptor in the IC may be associated with axosomatic synapses, although the contribution of axodendritic synapses to the presynaptic function of GABA_B_ receptors in the ICd cannot be ruled out. Moreover, it's very possible that spillover of GABA from the synaptic cleft can activate the postsynaptic GABA_B_ receptors in the neuropil in the ICd. Further experiments have yet to be conducted to confirm these suggestions.

### Auditory functions of the GABA_B_ receptor

Previous *in vivo* neurophysiological studies have shown that responses elicited by tone bursts and amplitude modulated tones in the IC can be changed by local application of agonists or antagonists of the GABA_B_ receptor (Faingold et al., 1989; Szczepaniak and Møller, 1995, 1996; Vaughn et al., 1996; Burger and Pollak, 1998). These results indicate that the GABA_B_ receptor is involved in auditory processing in this structure. It has yet to be determined what specific aspects of auditory processing are dependent on the GABA_B_ receptor.

As the GABA_B_ receptor has multiple pre- and postsynaptic functions including the mediation of inhibitory postsynaptic potentials and regulation of the release of glutamate and GABA, the receptor likely plays an important role in many aspects of auditory processing in the IC. However, it is unlikely that these functions are related to the processing of fine temporal structures of sounds. The reason is that the time course of the activation of the receptor is relatively slow, as this activation results in changes in the opening probability of ion channels through involving a guanine nucleotide-binding protein and multiple intracellular signaling steps (Chalifoux and Carter, 2011). In the IC, the duration of the inhibitory postsynaptic current mediated by the GABA_B_ receptor lasts for over 800 ms (Sun and Wu, 2009).

The pre- and postsynaptic functions of the GABA_B_ receptor along with the slow time course of activation suggests that the receptor is important for regulating the overall neural sensitivity to sounds as well as for setting the gain of signal processing in auditory neurons. Long-lasting inhibition mediated by the GABA_B_ receptor can counteract with long-lasting excitation mediated by the NMDA receptor. This counteraction can help maintain a balance between excitation and inhibition in neural processing (Morrisett et al., 1991; Sun and Wu, 2009).

The GABA_B_ receptor is important for plastic changes of neural sensitivity to stimuli in the IC. *In vitro* neurophysiological recordings in the ICc have revealed that presynaptic GABA_B_ receptors contribute to long-term potentiation in the structure (Zhang and Wu, 2000). Studies in other auditory structures as well as non-auditory structures have shown that GABA_B_ receptors also contribute to other forms of plastic changes of neural responses. For example, postsynaptic GABA_B_ receptors in the lateral superior olive are important for long-term depression of inhibitory transmission (Kotak et al., 2001; Chang et al., 2003). Presynaptic GABA_B_ receptors are important for short-term depression of glutamatergic transmission in avian auditory brainstem neurons (Brenowitz et al., 1998) and frequency-dependent depression of excitatory potentials in perirhinal cortex (Ziakopoulos et al., 2000). It has yet to be determined whether GABA_B_ receptors in the rat's IC can also cause these types of plastic changes in synaptic responses. In response to sounds, neurons in the IC display a type of short-term plastic change termed stimulus-specific adaptation (SSA) (Pérez-González et al., 2005, 2012; Malmierca et al., 2009a; Lumani and Zhang, 2010; Patel et al., 2012). These neurons reduce their sensitivity to sounds over repetitive presentations and increase the sensitivity when novel sounds are presented in the acoustic environment. Among the three subdivisions of the IC, the ICd and ICx have more neurons showing high-degree of SSA. In the ICd, the generation of SSA is likely dependent on inhibitory events that are slightly delayed but long lasting compared with excitatory events (Patel et al., 2012). The relatively slow time course of activation of the GABA_B_ receptor makes it an ideal candidate for mediating these long-lasting inhibitory events. The coincidence between strong SSA in the ICd and a high level of the GABA_B_ receptor in this collicular subdivision supports the involvement of the receptor in the generation of SSA. *In vivo* neuropharmacological experiments have yet to be conducted to verify this possibility.

The level of sensitivity to sounds and the gain of signal processing in auditory neurons are important for many other aspects of hearing. Age-related hearing loss, tinnitus, and audiogenic seizures can all be related to an abnormal level of sensitivity and gain in the IC (Caspary et al., 1995; Faingold, 2002; Wang et al., 2011). With its pre- and postsynaptic functions in regulating the sensitivity and gain in auditory neurons, the GABA_B_ receptor has to maintain a normal level of expression in the IC for preventing these hearing problems from occurring.

### Conflict of interest statement

The authors declare that the research was conducted in the absence of any commercial or financial relationships that could be construed as a potential conflict of interest.
